# Audit of Microalbumin Excretion in Children with Type I Diabetes

**DOI:** 10.4008/jcrpe.v1i3.44

**Published:** 2009-02-04

**Authors:** Filiz Mine Çizmecioğlu, Kathryn Noyes, Louise Bath, Chris Kelnar

**Affiliations:** 1 Department of Pediatrics, Endocrinology and Diabetes Unit, University of Kocaeli, Turkey; 2 Paediatric Diabetes Department of Diabetes, Royal Hospital for Sick Children, Edinburgh, UK; 3 Department of Paediatric Endocrinology, Royal Hospital for Sick Children, Edinburgh, UK; 4 Professor in Paediatric Endocrinology Department of Diabetes, University of Edinburgh and Royal Hospital for Sick Children, Edinburgh, UK; +90−532 788 43 65+90−262 303 80 03filizcizmeci@gmail.comKocaeli University Faculty of Medicine Umuttepe, 41380, Kocaeli, Turkey

**Keywords:** type 1 diabetes, Microalbuminuria, children and adolescents

## Abstract

**Objective**: To investigate prevalence, persistence and clinical correlates of increased microalbumin excretion in random urine samples collected in a paediatric diabetes clinic.

**Method**: Random urine samples were collected annually in patients >10 years attending the diabetes clinic in the Royal Hospital for Sick Children, Edinburgh. Albumin excretion is expressed as albumin:creatinine ratio (ACR) and classified as normal (10mg/mmol), or macroalbuminuria (>47 mg/mmol in females, >35 mg/mmol in males). We analyzed retrospectively results on 421 urine samples collected from 217 patients (109 males), of a median age of 12.3 years (94% 10−16 years) over 3 years. For each sample, the corresponding mean HbA1c over the previous year was calculated.

**Results**: Prevalence of micro− and macro−albuminuria in individual samples was 1% and 0.5% respectively. ACR was equivocal in 10.1% and 4.7% in samples from females and males respectively (p=0.03). HbA1c showed borderline significant differences across ACR groups (p=0.06). Equivocal ACR excretion was associated with slightly higher mean HbA1c (9.5±1.3%) compared to normal albuminuria (9.0±1.1%, p3.5 mg/mmol. The 14−16 years age group patients were most likely to have ACR >3.5 mg/mmol (p=0.05).

**Conclusions**: Female sex and increasing age, but not HbA1c, were independently associated with increased ACR. A robust mechanism for collection of repeat early morning urine samples from patients with increased ACR in random urine samples, and follow−up of those patients who have persistently high microalbumin excretion are important. It is also important  to confirm the usefulness of ACR measurements in random urine samples as a marker of incipent nephropathy.

**Conflict of interest:**None declared.

## INTRODUCTION

Age, sex, duration of the diabetes, glycemic control, blood pressure and genetic susceptibility are considered as risk factors which influence the development of early nephropathy in childhood type 1 diabetes.(1) Early detection of a rise in albumin excretion while still within the normal range would enable intervention to prevent the development of incipient nephropathy.(2, 3, 4) Therefore, annual screening of albumin excretion in urine for early detection of microalbuminuria (MA) is both necessary and useful, especially in children with diabetes of a duration greater than 10 years. This screening requires careful collection of the urine sample. Accurately timed urine is necessary to determine albumin excretion rate (AER) and this is considered as the gold standard in urine collection. However, when this is not possible, determination of albumin/ creatinine ratio (ACR) in early morning urine (EMU) or in random urine specimens are other alternatives which can be used for detection of MA.(5)

There is an unequivocal relationship between poor glycemic control and the development of MA.(6) However, independent of poor glycemic control, early diabetic complication risk increases with the onset of puberty. In addition, compared with male patients, MA risk is twofold greater in pubertal female patients.(1, 7) This is in contrast to lifetime risk of diabetic nephropathy, which is greater in male patients.(8) Diabetes Control and Complication Trial (DCCT) data showed that despite intensive insulin therapy, MA and elevated HbA_1c_ prevalence was higher in adolescents than in adults.(9)

This study was designed to investigate the prevalence and persistence of increased microalbumin excretion in random urine samples collected in a paediatric diabetes clinic. We also analysed MA risk based on clinical correlates such as age, sex and HbA_1c_.

## SUBJECTS AND METHODS

**Figure 1:**
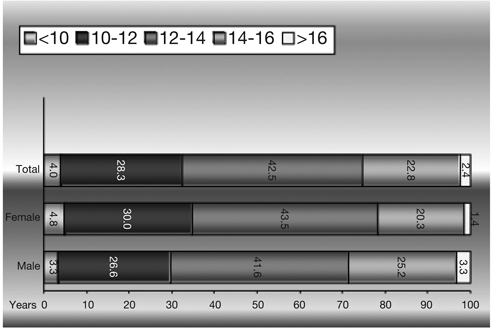
Sample based age distribution

All patients attending the diabetes clinic in the Royal Hospital for Sick Children (RHSC), Edinburgh for 3 years were evaluated retrospectively for MA. MA was measured in terms of ACR in random urine samples annually in all patients. Albumin was measured by turbidimetry on the Olympus analyser. Creatinine was measured by the Jaffe method. HbA_1c_ was measured by an immunological method on the DCA 2000 analyser, at least four times a year and mean HbA_1c_ for the previous year was calculated for each patient from these measurements. MA results in 23 urine samples did not match HbA_1c_ results and were excluded. In addition, unnecessary repeat urine samples (n=12) and dilute urine samples (n=7) were also excluded. A urine sample was considered too dilute if the creatinine was so low that it was not possible to determine whether the albumin excretion was normal or abnormal. The final number of urine samples for MA measurements included in the study was a total of 421, with corresponding HbA_1c_ measurements, from 217 diabetic patients. 108 of these patients were females (49.8%) and 109 (50.2%) were males. Age and sex information for each patient wasobtained from the hospital database. There were only few samples from patients younger than 10 years and older than 16 years. The age distribution was similar for males and females (Figure 1).

Microalbumin was measured in terms of ACR in random urine samples and categorized as follows:(10)

• <3.5 mg/mmol → Normoalbuminuria

• 3.5−10 mg/mmol → Equivocal

• >10 mg/mmol → MA

• >47 mg/mmol → Macroalbuminuria

If ACR showed normoalbuminuria, no further samples were collected until the next annual follow−up. If ACR exceeded normoalbuminuria or if the urine was so dilute that it was not possible to evaluate microalbumin excretion, a repeat sample was collected at the next clinic visit. If this repeat sample also showed equivocal or increased microalbumin excretion, the patient was instructed to collect an EMU. If the EMU showed normoalbuminuria, it was assumed that any previous equivocal or increased microalbumin excretion was due to orthostatic/exercise effects. Patients with equivocal or increased microalbumin excretion did not receive specific treatment other than advice regarding diabetes control and continued to be monitored at annual intervals.

MA data included many samples for which microalbumin was <5 mg/L with varying creatinine concentrations, resulting in normal (<3.5 mg/mmol) but not precisely quantifiable microalbumin normalised to creatinine. For statistical analysis, a result of, for example, <3.5 mg/mmol was expressed as 3.1 mg/mmol.

Patients were grouped by MA categories and by age. By microalbumin categories, group I designates normoalbuminuria, group II patients with equivocal results, and group III MA and macroalbuminuria. Age categories included five groups: <10, 10−12, 12−14, 14−16, and >16 years (Figure 1).

**Statistical Analysis**

We used both SPSS and Eviews for statistical analysis. Summary statistics are given as mean ± SD and/or median. Comparison of boys versus girls were based on two−sample t−test statistics. Pairwise equality of the means as well as equality of group means collectively were compared using Wald test based on χ^2^−statistic to overcome small sample bias for some groups.

To test the effects of age, sex and mean HbA_1c_ on the MA risk level, multiple regression analysis was used. Furthermore, controlling for the effects of sex and HbA_1c_, another multiple regression was run to detect which age group was at a higher risk level. All p−values in both regressions were calculated according to χ^2^−statistic. Dummy variable multiple regression method was used allowing to test the pairwise or collective equality of estimated regression coefficients which show that mean value of each variable under consideration across specific categories such as age and sex.

## RESULTS

Sample−based average age was 12.4 ± 1.2 (median 12.3 with a range of 1.1−22.2) years. Both the sample−based and patient−based distributions across sex were equal (female patients and samples: 49.8% and 49.2%, male patients and samples: 50.2% and 50.8%).

Mean HbA_1c_ was 9.07 ± 1.1% (median 9, range 6.3−12.8) and MA value 2.39 ± 9.15 mg/mmol (median 1.1, range 0.1− 175.4) in the whole sample. HbA_1c_ mean for females was 9.1 ± 1.1% and for males 9.0 ± 1.0%. Median HbA_1c_ value was 9% for both females and males. Mean MA value of females (3.1 ± 12.8 mg/mmol) was not statistically different (p=0.13) from mean MA of males (1.7 ± 2.3 mg/mmol). Median MA was 1.2 mg/mmol and 1 mg/mmol for males and females, respectively.

**Figure 2:**
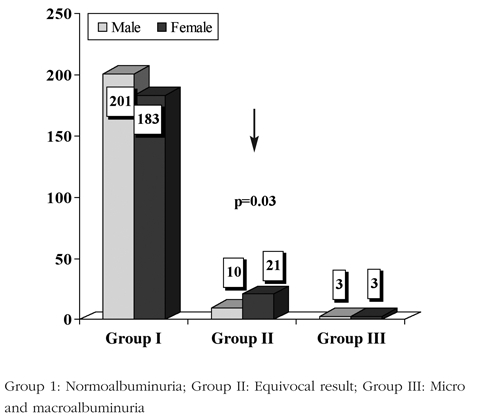
Gender differences across the MA groups

The prevalence of MA was found to be 1% while prevalence was 0.5% for macroalbuminuria. In 7.4% of the samples (10.1% of samples from females and 4.7% of those from males, p=0.03) ACR results were equivocal (Figure 2).

Using Wald test based on χ^2^−statistic, it was also tested whether there is any difference with respect to sex within each microalbumin category. There was a statistically significant difference regarding sex in group I (males > females; p=0.04) and group II (males < females; p=0.03, i.e., proportionately more females than males had equivocal albumin excretion) and no significant difference between females and males in group III.

There were 37 non−normal samples (8.8%) of which 31 were equivocal, 4 MA, and 2 macroalbuminuria from a total of 74 samples from 32 patients (21 females, 11 males). The clinical and biochemical features of patients with micro−and−macroalbuminuria are shown in Table 1.

There were 25 patients for whom follow− up samples were available. Six patients either remained in the same category or showed inconsistent fluctuations. 8 of 10 patients who improved were females. Spontaneous regression and progression rates were 40% and 36%, respectively.

There was no overall significant difference among microalbumin groups with respect to age (p=0.13). HbA_1c_ showed borderline significant differences across ACR groups (p=0.06).

Equivocal ACR excretion was associated with slightly higher HbA_1c_ (9.5±1.3%) than normal excretion (9.0±1.1%, p<0.05) (Table 2).

Multiple regression modelling indicated that increasing age (p=0.02), female sex (p=0.05) but not HbA_1c_ (p=0.79) were associated with a greater likelihood of ACR >3.5 mg/mmol in random urine samples. The 14−16 year age group were most likely to have ACR >3.5 mg/mmol (p=0.05) (Table 3).

**Table 1:**
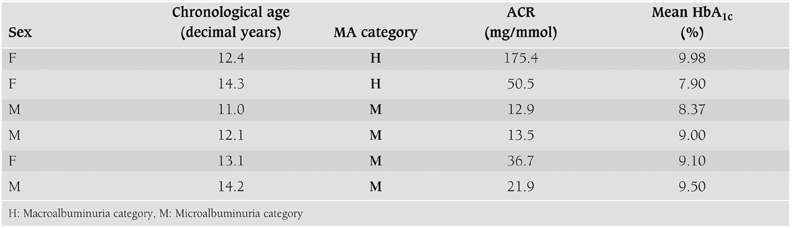
Clinical and biochemical features of patients with micro and macroalbuminuria

**Table 2:**
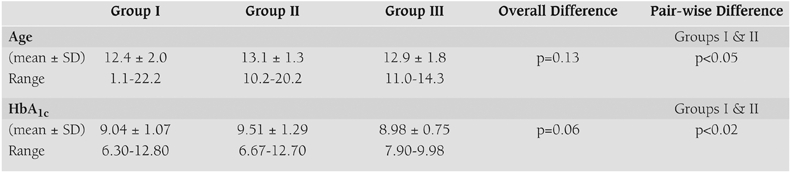
Comparison of age and HbA_1c_ across microalbuminuria groups

**Table 3:**
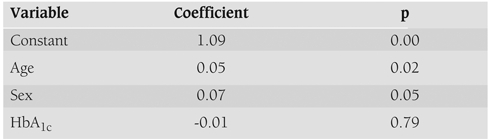
The association of MA risk with age, sex and HbA_1c_

## DISCUSSION

Diabetic nephropathy is a leading cause of increased morbidity and mortality in patients with type 1 diabetes. MA is an early predictor for diabetic nephropathy and is rarely detected before puberty. Its prevalence in adolescence varies from 4% to 20%, depending on the patient population and the screening method.(11, 12, 13) Annual ACR control in random urine is useful as a screening for the early detection of MA.(10, 14) If and when necessary, EMU can be requested as a second step. It is suggested that multiple samples should be taken to minimise biological variation. As a rule of thumb, ACR is above the cut−off value in two thirds of total EMU samples.(5)

According to our results, the prevalence of elevated ACR was 9% in our group of diabetic children. In the Danish study, this ratio was 13%. The youngest patient with MA was 15.3 years old in the Danish study whereas 11 years old in our study. While the oldest patient with macroalbuminuria was 19.1 years old in Danish study, it was 14.2 in our study. The onset of MA and macroalbuminuria was earlier in our study. Mean HbA_1c_ in the Danish cohort study was 9.7% and only a few patients had values below 8%.(12) Our mean HbA_1c_ was 9% with no significant difference between females and males. Fifteen percent of the HbA_1c_ samples showed values below 8%.

Both sample−based and patient−based distributions of females and males in all microalbumin groups were very similar. However, equivocal samples from female patients were twice higher than males. Overall, females had higher MA values in micro−and−macroalbuminuric groups. Consistent with previous literature,(7, 15, 16) our results also indicate that especially adolescent females with diabetes are at a higher MA risk compared to males.

MA risk increases also with age.(1) The Danish study showed an increasing prevalence with age, reaching 14% by 17−18 years.(17) Patients with equivocal ACRresults should be followed up closely considering that they are within the grey zone, especially starting with the onset of puberty.

It is well known that poor glycemic control leads to an increase in diabetic complications. In our study, it was surprisingly found that glycemic control did not appear to influence MA risk. In the Danish experience, MA prevalence was related to HbA_1c_ only in girls.(17) In other studies, it is reported that MA is unequivocally related to poor glycemic control, but that the increasing prevalence of MA during puberty cannot be entirely explained by HbA_1c_.(7) These findings raise a question on the possibility of potential factors for MA risk other than HbA_1c_, especially in females in the pubertal period. Transient insulin resistance during puberty may have a role to development of MA. Amin et al(7) pointed out the link between hormonal changes (hyperandrogenism, low−sex hormone binding globulin [SHBG], insulin like growth factor [IGF1] levels) and MA in female adolescent patients. Hormonal changes during puberty may effect the development of MA especially in susceptible girls. The development of MA at puberty may reflect not only poor glycemic control but also changes in the growth hormone−IGF1 axis and ovarian function.(7)

DCCT data, on the other hand, demonstrate that hyperglycaemia plays a prominent role in development of microvascular complications in adolescents. Aggressive insulin therapy has been shown to reduce diabetic complication risk.(6) However, this may confer increased weight gain and hypoglycaemia and predispose the paient to the detrimental effects of peripheral hyperinsulinemia, such as the development of ovarian hyperandrogenism.(7) We also know that puberty is characterised by relative insulin resistance,(18) and near−normoglycaemia may be difficult to achieve in this period.12 Insulin resistance can predate the appearance of MA.(19) Ovarian hyperandrogenism in polycystic ovarian syndrome is related to insulin resistance and peripheral hyperinsulinemia.(20) The GH−associated increase in insulin resistance during puberty in type 1 diabetes may be the principal cause of the ovarian hyperandrogenism.(7) 

However, the relationship between MA and blood pressure is still controversial. It has been claimed that blood pressure is not an important initiating factor for MA in children with type 1 diabetes.(14, 21)

In conclusion, female sex and increasing age, but not HbA_1c_, were independently associated with increased ACR in random urine samples from adolescents. Orthostatic and other factors unrelated to glycemic control may have contributed to these findings.

Patients with equivocal ACR−results should be followed up closely, especially after the onset of puberty. Given that there was no significant relation between MA risk and HbA_1c_ in our study, further research is needed to completely clarify this relationship, especially in females in the pubertal period.

A robust mechanism for collection of repeat early morning urine samples from patients with increased ACR in random urine samples, and follow−up of those patients who have persistently high microalbumin excretion are important in the care of diabetic patients. It is also important to further confirm the usefulness of ACR measurements in random urine samples as a marker of incipent nephropathy.

## ACKNOWLEDGEMENT

This study was carried out during the 3 months period spent in Edinburgh Sick Kids Hospital in Scotland, in accordance with the European Society of Pediatric Endocrinology (ESPE) Clinical fellowship programme. We express our gratitude to the laboratory where the microalbumin measurements were carried out: Department of Clinical Biochemistry, Royal Infirmary, Edinburgh. We are grateful to Dr.Patricia M. Crofton from Department of Paediatric Biochemistry, Royal Hospital for Sick Children, Edinburgh, UK. We would like to thank Ercan Balaban and Charalambos Constantinou from the University of Edinburgh for their contribution to the statistical analyses. We also thank for the helpful contribution of Prof. Feyza Darendeliler from Istanbul University in reviewing the manuscript.
